# Analysis of T follicular and T peripheral helper lymphocytes in autoimmune thyroid disease

**DOI:** 10.1007/s12020-024-03686-7

**Published:** 2024-06-15

**Authors:** Raquel Sánchez-Gutiérrez, Rebeca Martínez-Hernández, Ana Serrano-Somavilla, Miguel Sampedro-Nuñez, Alejandra Mendoza-Pérez, José Luis Muñoz de Nova, Marlen Vitales-Noyola, Roberto González-Amaro, Mónica Marazuela

**Affiliations:** 1 Section of Molecular and Translational Medicine, CICSaB, UASLP, 78210 San Luis Potosí, SLP México; 2 Service of Endocrinology, Hospital Universitario de La Princesa, Instituto de Investigación Princesa, Universidad Autónoma de Madrid, Madrid, España; 3 Department of Immunology, School of Medicine, UASLP, 78210 San Luis Potosí, SLP México

**Keywords:** Tfh cells, Tph cells, Plasmablast, PD-1, Autoimmune Thyroid Disease

## Abstract

**Purpose:**

Peripheral helper T (Tph) cells have an important role in the induction of humoral immune responses and autoantibody production. Accordingly, it is feasible that this lymphocyte subset has a relevant role in the pathogenesis of autoimmune thyroid diseases (AITD). In this study we aim to analyze the levels and function of Tph cells in blood samples from patients with AITD.

**Methods:**

We performed an observational study with cases and controls. Blood samples were obtained from nineteen patients with Hashimoto’s thyroiditis (HT), twenty-four with Graves’ disease (GD), and fifteen healthy controls. In addition, the levels of follicular T helper (Tfh) cells and Tph cells, the release of interleukin-21 (IL-21) by these lymphocytes and the number of plasmablasts were analyzed by multi-parametric flow cytometry analyses.

**Results:**

Increased percentages of Tfh and Tph lymphocytes were detected in patients with HT and GD. Furthermore, an enhanced synthesis of the cytokine IL-21 by these cells was observed. Accordingly, we detected significant higher percentages of plasmablasts in patients with GD, and these values tended to be also higher in HT patients. Moreover, significant positive associations were observed between the levels of Tfh or Tph and the number of plasmablast or anti-TSHR Ab titers in patients with AITD.

**Conclusion:**

Our data suggest that Tph lymphocytes may have a relevant role in the pathogenesis of AITD.

## Introduction

Autoimmune thyroid disease (AITD) is a common organ specific disorder characterized by immune reactivity against self-thyroid antigens [1]. In addition, these patients show abnormal levels and function of different lymphocyte subsets, including T regulatory (Treg) cells, Th17/Th22 lymphocytes and NK cells [2–6]. In this regard, and as it has been widely described, Graves’ disease (GD), is an autoimmune disorder that mainly affects the thyroid gland and that is characterized by an excessive synthesis of thyroid hormones. The pathogenesis of GD involves the breakdown of immune tolerance towards thyroid self-antigens, leading to the activation of B lymphocytes and the development of a humoral autoimmune response. The thyroid stimulating hormone receptor (TSHR) is the main target antigen of the immune system in GD, which induces the synthesis of autoantibodies against it, stimulating the secretion of thyroid hormones and leading to gland hyperplasia and hyperthyroidism [1]. In contrast, Hashimoto’s thyroiditis (HT) is mainly characterized by the induction of a cellular autoimmune response directed against different thyroid antigens and mainly mediated by Th1 and Th17 lymphocytes. This autoimmune phenomenon leads to a destructive inflammatory process in the gland, causing hypothyroidism. However, one additional important feature of HT patients is the presence of increased levels of autoantibodies directed against thyroperoxidase (anti-TPO Ab) and thyroglobulin (anti-TG Ab), indicating that a humoral autoimmune response and the synthesis of autoantibodies also participate in the pathogenesis of this condition [1, 7].

Follicular T helper cells (Tfh) are a subset of CD4^+^ T lymphocytes mainly located in the germinal centers of secondary lymphoid organs (SLO), which are formed mostly by B lymphocytes [8]. Tfh cells play a key role in providing help for naïve B cells undergoing their differentiation into plasmablasts or memory B cells [8, 9]. Thus, Tfh cells induce the maturation and proliferation of B cells, increasing antibody production. Accordingly, different studies strongly suggest that Tfh lymphocytes may have a relevant role in autoimmune diseases mediated by auto-antibodies, including AITD [10–14].

Several reports have contributed to define the phenotype of Tfh cells. In this regard, it has been informed the expression of the chemokine receptor CXCR5 by these cells, which directs their migration towards the germinal center [9, 15]. Furthermore, the expression of the programmed death protein 1 (PD-1, CD279) as well as the inducible T cell co-stimulator (ICOS, CD278) by these cells have been also reported [16]. In addition, Tfh cells are characterized by the production of interleukin 21 (IL-21), a cytokine that plays a pivotal role in B cell differentiation into plasma cells and the formation and maintenance of germinal centers into secondary lymphoid tissues [16, 17]. According to this, it is now feasible to accurately detect and quantify Tfh cells by using multiparametric flow cytometry.

An additional subset of CD4^+^ T cells closely related to Tfh lymphocytes, and also showing the ability to provide significant help to B cells, has been described in the peripheral blood and synovial fluid from patients with rheumatoid arthritis [18]. These Tph or peripheral helper T cells, in contrast to Tfh cells, do not express the chemokine receptor CXCR5; however, the CCR2, CCR5 and CX3CR1 receptors are detected on their cell surface [18, 19]. Furthermore, these cells, as their counterparts located into the germinal centers, synthesize large amounts of IL-21 [20]. In this regard, several phenotypes have been employed for the flow cytometry analysis of this lymphocyte subset under different conditions. Accordingly, Rao et al. defined the Tph cell phenotype as CD4^+^CXCR5^-^PD-1^hi^CD278(ICOS)^+^ plus the expression of CD38 and CD69 [18]. However, other groups have employed only three markers (CD4^+^CXCR5^-^PD-1^hi^) for its analysis, mainly in samples from patients with autoimmune diseases [11, 20, 21]. In addition, in a recent study, these cells were simply defined as CD4^+^CXCR5^-^PD-1^+^, without considering the level of expression of PD-1 [22]. Although several studies have assessed the levels of Tfh cells in samples from patients with AITD [10, 12], to our best knowledge Tph cells have not been hitherto analyzed in this condition.

In this study, we analyzed the levels of Tph, Tfh and plasmablast cells in the peripheral blood of patients with AITD and healthy controls. Furthermore, we evaluated the possible association between the levels of these cells and clinical parameters. We detected high levels of Tfh, Tph and plasmablast cells in AITD, which were associated with the titers of autoantibodies in patients with GD and HT. These data suggest that Tph lymphocytes are involved in the pathogenesis of thyroid autoimmunity.

## Materials and methods

### Individuals and samples

Blood samples were obtained from forty-three patients with AITD, nineteen with HT and twenty-four with GD. Samples from fifteen healthy individuals were used as controls. Approximately 70% of patients were female, 30% male, with a mean age of 50 ± 14 years (arithmetic mean ± SD). Controls had a similar female to male proportion, mean age, and none of them showed any evidence of a thyroid disorder or an autoimmune disease. At the time of the study,17 patients with GD (70%) were on anti-thyroid therapy, and 11 patients with HT (58%) were receiving levothyroxine. All patients with AITD were examined by an endocrinologist, and the diagnosis was established on commonly accepted clinical, laboratory, and histological criteria for HT or GD [23, 24]. Main clinical and demographic data of patients included in the study are shown in Table 1, and a written informed consent was obtained from all of them. This study was approved by the Hospital Bioethical Committee (Hospital Universitario de La Princesa, Madrid, Spain).

**Table 1:** Clinical data of AITD patients

AITD	Patients	
	HT	GD
n	19	24
Gender (F/M)	15/4	16/8
Age (y)	54.5 ± 14	47.8 ± 12
Time of evolution	2.0 (1.0–5.0)	1.0 (1.0–2.25)
Treatment (yes/no)	11/8	17/7
TSH, µU/mL	4.24 (0.23–6.24)	0.01 (0.01–0.017)
T4, ng/dL	1.10 (0.99–1.42)	2.36 (1.86–4.13)
Tg-Ab, UI/mL	196 (20.25–660.3)	463 (20.50–1421)
TPO-Ab, UI/mL	617.0 (92.75–978.5)	223 (5.25–1319)
TSHR-Ab, U/L	0.62 (0.5–3.11)	3.15 (0.98–7.61)

Values show number of categorical values and median (interquartile intervals Q1–Q3) for continuous variables. TSH normal range = 0.27–4.20; T4 normal range = 0.93–1.7

*AITD* Autoimmune thyroid disease, *F/M* female-male, *GD* Graves disease, *HT* Hashimoto thyroiditis, *Tg-Ab* anti-thyroglobulin antibody (negative < 344), *TPO-Ab* anti-thyroid peroxidase antibody (negative < 100), *TSHR-ab* anti-thyrotropin antibody (negative < 0.7)

### Cell isolation

Peripheral blood mononuclear cells (PBMCs) from patients and controls were isolated by Ficoll-Hypaque (Sigma Chemical Co., St. Louis, MO) density-gradient centrifugation. Cells were maintained in RPMI 1640 (GIBCO, Grand Island, NY) supplemented with 10% fetal bovine serum (Hyclone, Logan, UT), penicillin (50 IU/mL), and streptomycin (50 μg/mL) (Sigma). Cell viability was assessed by trypan blue staining, and it was always greater that 95%.

### Flow cytometry analysis

For the analysis of the frequency of Tph and Tfh lymphocytes, PBMCs were stained with the following monoclonals antibodies (mAbs): anti-CD4-PerCP (BD Biosciences), anti-PD-1-Pacific Blue (BioLegend), anti-CXCR5-APC-Cy7 (BioLegend), anti-CD38-PE (eBiosciences), anti-CD69-APC (BioLegend), anti-ICOS(CD278)-FITC (Invitrogen), and anti-IL-21-APC (BioLegend). Plasmablasts were analyzed by using the following mAbs: anti-CD19-PerCP (BD Biosciences), anti-CD27-FITC (eBioscieces), and anti-CD20 APC-Cy7 (BD Biosciences). Then, cells were washed and resuspend for their analysis in a FACS Canto II flow cytometer with the FACS Diva Software (BD Biosciences). To set the gates, we used the Fluorescence Minus One strategy, leaving out one mAb (the one of interest) at a time (the opposite of single stain controls). In this strategy, Tfh cells were defined as CD4^+^PD-1^+^CXCR5^+^CD38^+^CD69^+^ICOS^+^, whereas Tph cells corresponded to CD4^+^PD-1^+^CXCR5^-^CD38^+^CD69^+^ICOS^+^. Moreover, CD19^+^CD27^+^CD20^-^CD38^hi^ cells were considered as plasmablasts.

### Intracellular cytokine staining

To assess the synthesis of IL-21 by Tph and Tfh cells, 1.5 × 10^6^ PBMC were cultured in flat bottom 24-well plates for 4 h, in the presence or absence of ionomycin (750 ng/mL) and PMA (50 ng/mL). Brefeldin A (10 μg/ml) was added in the last two hours of cell culture. Then, cells were harvested, labeled for CD4, PD-1, CXCR5, CD38, and ICOS, fixed with 1% p-formaldehyde, and permeabilized with 0.05% saponin. Finally, cells were stained with an anti-IL-21 mAb (eBiosciences) tagged with APC and analyzed by flow cytometry.

### Statistical analysis

Data are expressed as the arithmetic mean and SD for data with normal distribution, while median and interquartile range (Q_1_-Q_3_) for data with a non-Gaussian distribution. Comparisons between two groups were done with the Mann–Whitney U–test, whilst comparisons among three groups were analyzed with the Kruskal-Wallis sum rank test, and *post-hoc* analysis, if necessary. Association between two quantitative variables was analyzed with the Spearman or Pearson correlation tests, as required. Data were analyzed by using the GraphPad Prism software v5.0 (San Diego, CA) and *p* values < 0.05 were considered as significant.

## Results

### Patients with AITD show high levels of circulating Tfh and Tph cells

**Fig. 1:**
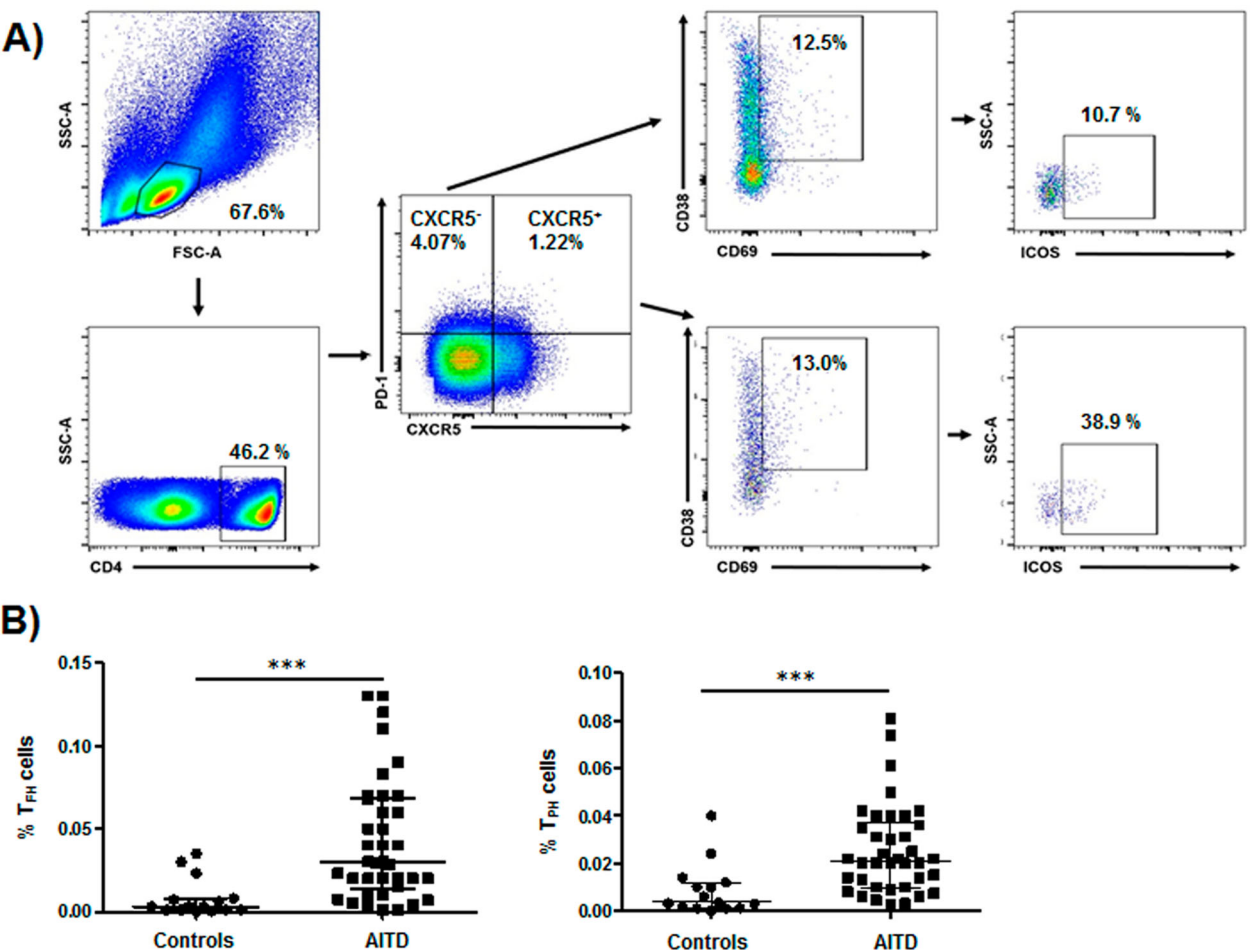
Multi-parametric flow cytometry analysis of Tfh and Tph cells in the peripheral blood from AITD patients and healthy controls. **A** Flow cytometry strategy for the analysis of Tfh and Tph cells. PBMC were labeled with the indicated mAbs and analyzed by flow cytometry, as stated in “Material and Methods”. Tfh lymphocytes were defined as CD4^+^PD-1^+^CXCR5^+^CD38^+^CD69^+^ICOS^+^, whereas Tph cells corresponded to CD4^+^PD-1^+^CXCR5^-^CD38^+^CD69^+^ICOS^+^. **B** Levels of the Tfh and Tph cell subsets in AITD patients and controls. Data correspond to the median and Q_1_-Q_3_ range. ***p < 0.001

**Fig. 2:**
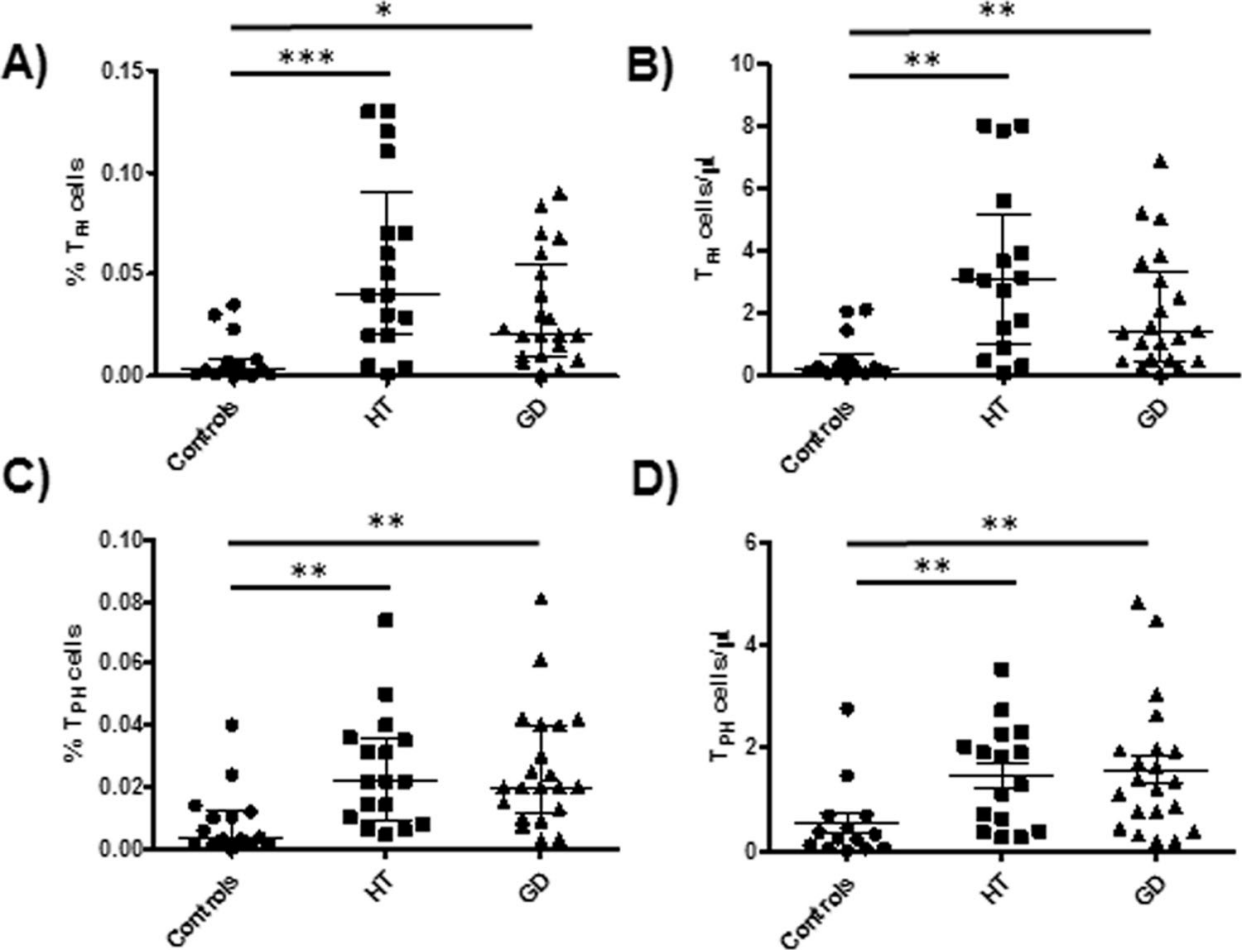
Levels of Tfh and Tph cells in patients with HT and GD and healthy controls. PBMC were labeled and analyzed by flow cytometry, as stated in “Material and Methods”. **A**, **C** Percent and absolute number of Tfh cells. **B**, **D** Percent and absolute number of Tph cells. Data correspond to median and Q_1_-Q_3_ range. *p < 0.05, **p < 0.01, ***p < 0.001

Tfh and Tph cells were analyzed by multi-parametric flow cytometry, according to strategy shown in Fig. 1A. In this regard, Tfh cells were defined as CD4^+^PD-1^+^CXCR5^+^CD38^+^CD69^+^ICOS^+^, whereas CD4^+^PD-1^+^CXCR5^-^CD38^+^CD69^+^ICOS^+^ lymphocytes corresponded to Tph cells. According to this analysis, we found that the percentage of Tfh lymphocytes was increased in patients with AITD compared to healthy controls (*p* = 0.0001, Fig. 1B). Likewise, an enhanced proportion of Tph cells were also found in patients with AITD compared to controls (*p* < 0.0001, Fig. 1B). In addition, we observed significant increased percentages and absolute numbers of Tfh cells in samples from patients with HT and GD compared to healthy controls (*p* = 0.0003 and *p* = 0.0003, respectively, Fig. 2A, B). When Tph lymphocytes were analyzed, both HT and GD patients showed a significant increased proportion and absolute number of these cells compared to controls (*p* = 0.0014 and *p* = 0.0028, respectively, Fig. 2C, D).

### High frequency of circulating plasmablast in patients with AITD

**Fig. 3:**
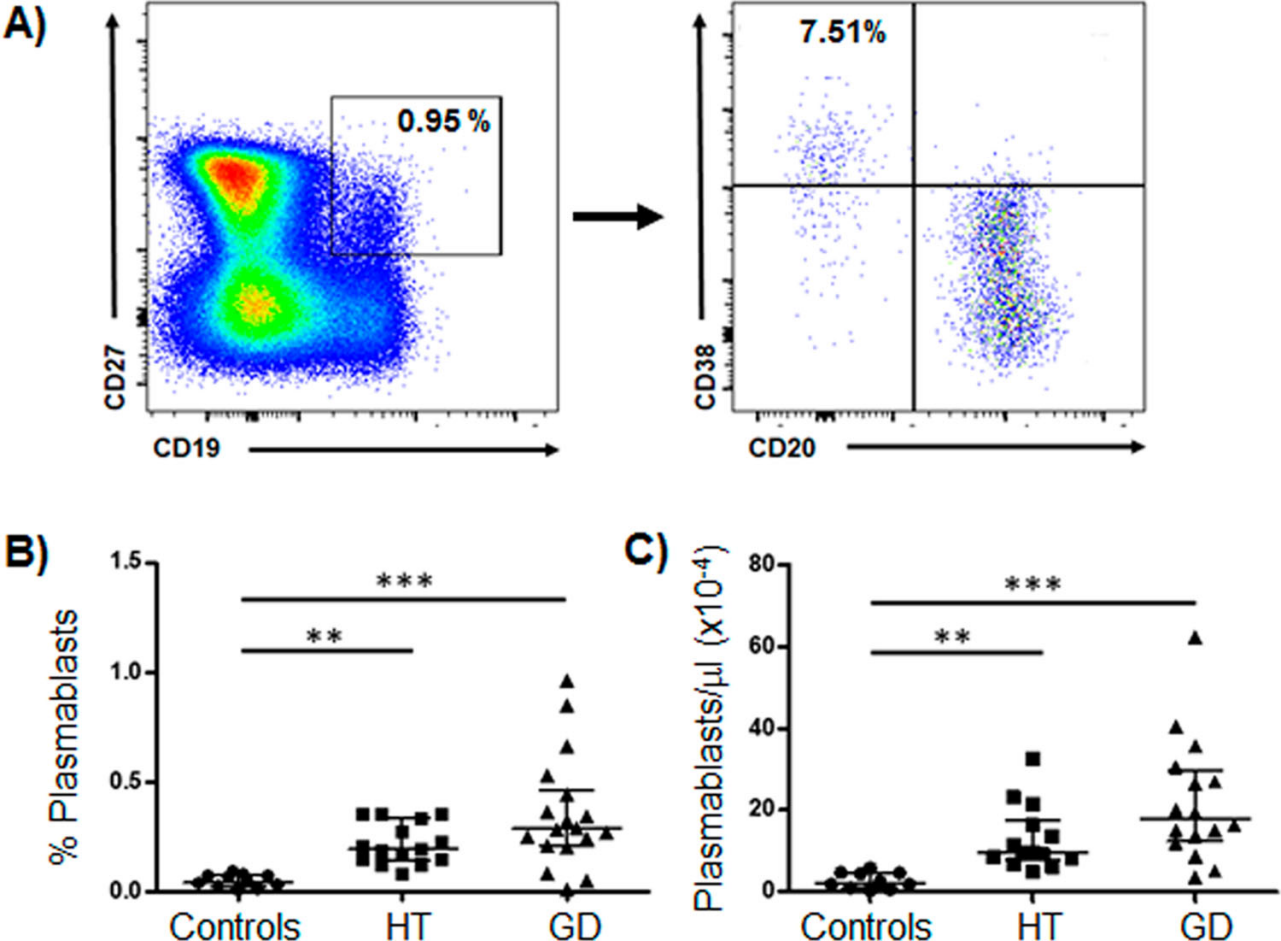
Flow cytometry strategy for the analysis of circulating plasmablast. PBMC were stained with the indicated mAbs and analyzed by flow cytometry; CD19^+^CD27^+^CD20^-^CD38^hi^ were considered as plasmablasts. **A** Representative dot plot images of a plasmablast analysis are shown. **B**, **C** Percent and absolute number of plasmablasts in samples from patients with HT and GD and healthy controls. Data correspond to the median and Q_1_-Q_3_ range. **p < 0.01, ***p < 0.001

As shown in Fig. 3A, levels of plasmablast (CD19^+^CD27^+^CD20^-^CD38^hi^) were analyzed in peripheral blood samples by flow cytometry. In this analysis, we found a high frequency and absolute numbers of plasmablast in HT and GD patient compared to controls (*p* < 0.0001 in both cases, Fig. 3B, C). Accordingly, no significant differences in the level of these cells were detected between patients with HT and GD (*p* > 0.05 in both cases, Fig. 3B, C).

### Increased production of IL-21 by Tfh and Tph cells in patients with AITD

**Fig. 4:**
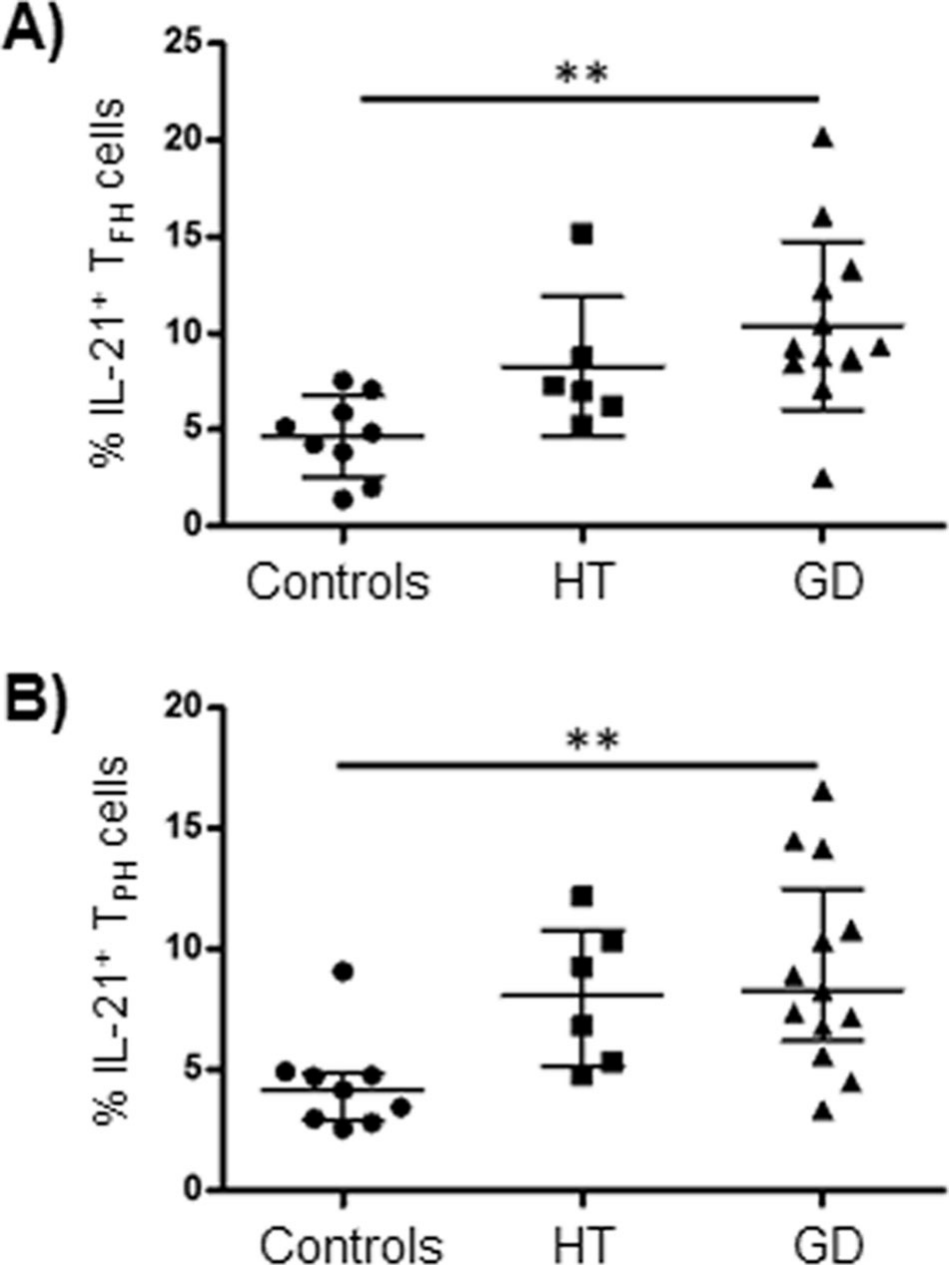
Analysis of the synthesis of IL-21 by PD-1^+^ Tfh and Tph cells from AITD patients and healthy controls. PBMC were stimulated with PMA and ionomycin, labeled with mAbs, permeabilized and incubated with an anti-IL-21 conjugated with APC. Then, cells were analyzed by flow cytometry, as indicated in “Materials and Methods”. **A** Percentages of Tfh lymphocytes expressing IL-21. **B** Percentages of Tph cells expressing IL-21. Data correspond to median and Q_1_-Q_3_ range. **p < 0.01

As shown in Fig. 4A, the percentage of IL-21^+^ Tfh cells was increased in GD (*p* = 0.0047, compared to controls). Similar results were observed in the case of IL-21^+^ Tph cells (*p* = 0.0064, Fig. 4B). When data were analyzed as the mean of fluorescence intensity of IL-21 staining, similar enhanced levels were observed in HT and GD samples compared to controls (*p* < 0.05 in both cases, data not shown).

### Levels of Tfh and Tph cells are associated with autoantibodies titers

**Fig. 5:**
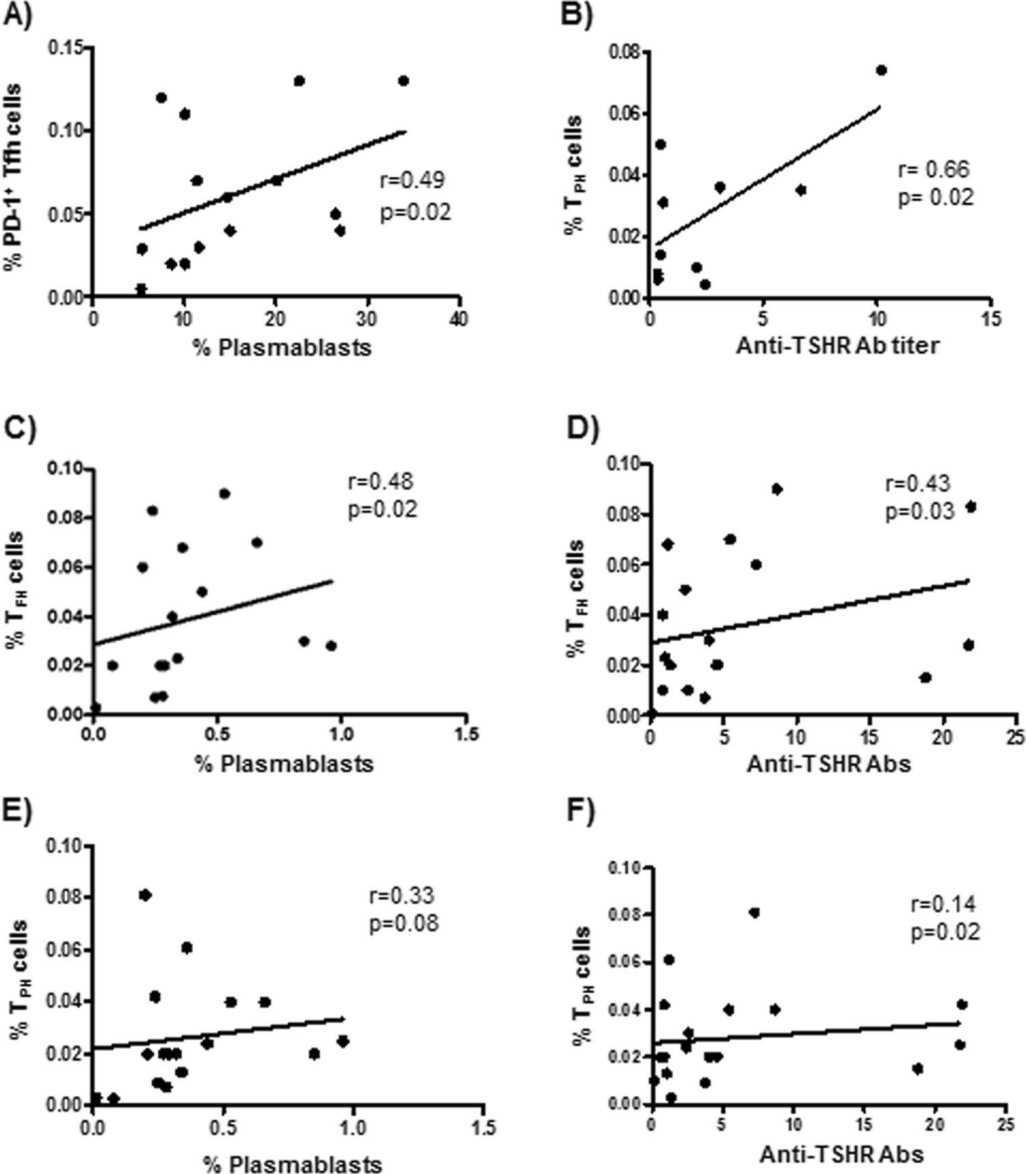
Correlation analysis of clinical laboratory parameters and percentages of Tfh and Tph lymphocytes in patients with AITD. **A** Correlation between the levels of Tfh cells and plasmablasts in patients with HT. **B** Association analysis between Tph cells and anti-TSHR Ab titers in HT patients. **C**, **D** Correlation between Tfh cells and frequency of circulating plasmablast or anti-TSHR Ab titers in patients with GD. **E**, **F** Correlation of Tph cells and plasmablast levels and anti-TSHR Ab titers in in patients with GD. Data were analyzed by using the Spearman rank correlation test. r and *p* values are indicated

As shown in Fig. 5A, a positive and significant correlation was observed between the percent of Tfh cells and plasmablast levels in patients with HT (r = 0.49, *p* = 0.029). A similar significant association was detected in GD patients between the percent of Tph cells and anti-TSHR Ab titers (r = 0.66, *p* = 0.02, Fig. 5B). Furthermore, in GD patients the proportion Tfh cells showed a positive significant association with the frequency of circulating plasmablasts (r = 0.48, *p* = 0.02, Fig. 5C) or the anti-TSHR Ab levels (r = 0.43, *p* = 0.03, Fig. 5D). Likewise, Tph cells tended to show a positive association with the number of plasmablasts (r = 0.33, *p* = 0.08, Fig. 5E) or anti-TSHR Ab titers (r = 0.14, *p* = 0.02, Fig. 5F). In addition, the levels of PD-1^high^ Tfh or Tph cells were significantly associated with anti-TPO and anti-TSHR ab titers (*p* < 0.05 in all cases, data not shown). Finally, a significant inverse association between the levels of PD-1^high^ Tfh cells and TSH concentrations was detected in patients with GD (r = −0.45, *p* = 0.03, data not shown).

## Discussion

Although several studies have investigated the role of Tfh and Tph cells in different autoimmune diseases [18–22], the possible involvement of Tph cells has not been previously explored in AITD patients. Therefore, we decided to analyze the levels of these two T helper cell subsets in patients with AITD, and we have hypothesized that these patients would show an enhanced number and function of these lymphocytes in their peripheral blood.

We detected in patients with AITD increased levels (percentages and absolute numbers) of Tfh and Tph cells as well as an enhanced synthesis of IL-21 by these cells. Although the precise role of these abnormalities in the pathogenesis of AITD requires additional studies, we think that these results support the involvement of these cells in the onset or progression of this condition. In this regard, it has been described that the levels of Tph cells in peripheral blood are associated with disease activity in patients with systemic lupus erythematosus and ulcerative colitis [22, 25]. Moreover, it has been described that in patients with rheumatoid arthritis Tph lymphocytes are able to release, in addition to IL-21, Th1-type pro-inflammatory cytokines (such as interferon-γ, tumor necrosis factor-α and granulocyte/monocyte colony stimulating factor) [18, 20, 26, 27]. Therefore, it is feasible that these Tph lymphocytes may contribute to the pathogenesis of HT, a condition mainly mediated by conventional Th1 lymphocytes, and the cytokines release by them.

In contrast with other lymphocyte subsets, the expression of the immune-regulatory molecule PD-1 by both Tfh and Tph cells does not seem to be related to a repeated exposure to the antigens recognized by them, which favors cellular exhaustion. In this regard, our data suggest that Tph lymphocytes may contribute to the humoral autoimmunity seen in patients with HT, mainly characterized by the presence of anti-TPO and anti-thyroglobulin autoantibodies. Likewise, these cells could be also involved in the induction of synthesis of anti-TSHR antibodies, in patients with GD. In this regard, it is of interest that whereas Tfh cells only provide help to naïve B lymphocytes into the germinal centers of lymphoid tissue, Tph cells may localize into inflamed tissues, providing help to memory B cells that are infiltrating those tissues [18]. According to this, we have attempted to analyze the presence of Tph cells in thyroid tissue sections from patients with AITD, and our preliminary results indicate that in HT there is a scarce but significant number of Tph cells into the inflammatory cell infiltrate (see Supplementary Fig. 1). However, as it has been previously discussed, the detection of Tph cells in tissue sections by conventional immunofluorescence microscopy is a difficult issue, since the main distinctive feature of these lymphocytes (compared to Tfh cells) is the absence (not the presence of) a cell marker, the chemokine receptor CXCR5 [18]. Thus, we consider that it would be of interest to carry out an additional study obtaining enough thyroid tissue from AITD patients, to make cell suspensions for flow cytometry analysis.

Since Tph and Tfh cells are the main source of IL-21, a cytokine that has a key role in the differentiation of B lymphocytes into the antibody producing cells plasmablasts, we decide to analyze the possible association between the levels of Tph and Tfh producing this cytokine and the number of blood plasmablasts in the patients included in this study. In this regard, we have found that both Tfh and Tph cells from patients with AITD show an increased synthesis of IL-21, which is accompanied by enhanced levels of plasmablasts in their peripheral blood. We consider that these data further suggest that Tfh and Tph cells are involved in the induction of the production of auto-antibodies, which have an important role in different autoimmune diseases. Accordingly, different reports have shown altered levels of Tfh cells in patients with AITD, suggesting its involvement in the pathogenesis of this condition [10, 28]. Therefore, we consider of interest our novel data, regarding the levels and function of Tph cells in patients with thyroid autoimmunity, which suggest that these cells also may have a relevant role in the pathogenesis of AITD.

In summary, our data indicate that, in addition to abnormalities in the levels and function of Tfh cells, patients with AITD also show an enhanced number and function of Tph lymphocytes, which very likely also contribute to the pathogenesis of this condition. In this regard, a detailed study on the presence and function of these helper lymphocytes in thyroid tissue from AITD patients would add valuable information about this interesting point. Furthermore, since we studied a limited number of individuals in each group, it would be interesting to perform a larger study in the future. Likewise, it would be interesting to analyze Tph cells in samples of thyroid tissue of AITD patients.

## Supplementary Information

Supplementary Figure 1

Supplementary Figure 1 caption
