# Supplementary figures and images for: Analysis of T follicular and T peripheral helper lymphocytes in autoimmune thyroid disease
Source: Endocrine. 2024 Jun 15;86(2):699–706. doi: 10.1007/s12020-024-03686-7 (PMC11489195; doi:10.1007/s12020-024-03686-7)

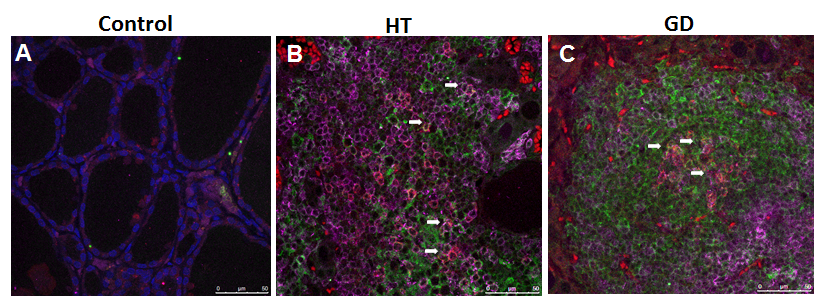

Supplement: Supplementary file 1 — Supplementary Figure 1 [file 12020_2024_3686_MOESM1_ESM.tif]
