# Supplementary material for: Analysis of T follicular and T peripheral helper lymphocytes in autoimmune thyroid disease
Source: Endocrine. 2024 Jun 15;86(2):699–706. doi: 10.1007/s12020-024-03686-7 (PMC11489195; doi:10.1007/s12020-024-03686-7)
Supplement: Supplementary file 2 — Supplementary Figure 1 caption [file 12020_2024_3686_MOESM2_ESM.docx]

**Supplementary Figures**

**Supplementary Fig. 1:** Representative images of control, HT and GD thyroid glands labelling Tph cells expressing CD4 (magenta) PD1 (red) and CCR2 (green) markers. Arrowheads show triple positive cells in thyroid infiltrating lymphocytes. Scale bar= 50μm.

Thyroid tissue sections from patients with HT were incubated overnight at 4 ᵒC with an anti-CD4 (R&D Systems), anti-PD1 and anti-CCR2 (Novus Biologicals) antibodies. Then, an Alexa 647 labelled donkey anti-goat, an Alexa 568 labelled donkey anti-mouse and an Alexa 488 labelled donkey anti-rabbit (Thermo Fisher Scientific) were used as secondary antibodies, and cell nuclei were counterstained with 4′,6-diamidino-2-phenylindole (DAPI). Goiter samples were performed as controls. Sections were analyzed in a Leica TCS-SP5 confocal microscope (Leica Microsystems, Wetzlar, Germany).
